# New insights into fever phobia: a pilot qualitative study with caregivers and their healthcare providers

**DOI:** 10.1007/s00431-022-04704-4

**Published:** 2022-11-29

**Authors:** Federica Merlo, Ilaria Falvo, Maria Caiata-Zufferey, Peter J. Schulz, Gregorio P. Milani, Giacomo D. Simonetti, Mario G. Bianchetti, Marta Fadda

**Affiliations:** 1 Institute of Public Health, Faculty of Biomedical Sciences, Università della Svizzera Italiana, Lugano, Switzerland; 2 Sasso Corbaro Foundation, Bellinzona, Switzerland; 3 Department of Business Economics, Health and Social Care, University of Applied Sciences and Arts of Southern Switzerland, Manno, Switzerland; 4 Faculty of Communication, Culture and Society, Università della Svizzera Italiana, Lugano, Switzerland; 5 Department of Communication & Media, Ewha Womans University, Seoul, Korea; 6 Pediatric Unit, Fondazione IRCCS Ca’ Granda Ospedale Maggiore Policlinico, Milan, Italy; 7 Department of Clinical Sciences and Community Health, Università degli Studi di Milano, Milan, Italy; 8 Pediatric Institute of Southern Switzerland, Ente Ospedaliero Cantonale, EOC, Bellinzona, Switzerland; 9 Faculty of Biomedical Sciences, Università della Svizzera Italiana, Lugano, Switzerland

**Keywords:** Fever phobia, Paediatrics, Parents, Paediatricians, Medical assistants, Healthcare providers, Infants, Qualitative

## Abstract

Despite the availability of clinical guidelines on the correct symptomatic management of fever in children, several studies have reported inaccurate knowledge about this symptom and inappropriate management behaviours among caregivers. There is evidence that caregivers’ management of fever is largely influenced by unrealistic and unwarranted concerns about the potential harm that elevated body temperature can cause, a phenomenon commonly referred to as fever phobia. Research on fever phobia has predominantly focused on the role of fever misconceptions in triggering anxiety and impeding a proper fever management, in terms of both concept and operationalization, with little attention to the influence of the relationship between caregivers and the healthcare team. The aim of this pilot study was to explore and describe fever-related knowledge, experience and behaviour among a sample of caregivers, paediatricians and their medical assistants in the Canton of Ticino, Switzerland. We used a qualitative study design with semi-structured, one-to-one interviews with paediatricians employed in private healthcare facilities, their medical assistants and caregivers with at least one child between the ages of 0 and 3 years. We conducted individual interviews either in person or by phone, according to participants’ preferences, between October 2020 and February 2021. We performed an inductive–deductive analysis of the transcripts to identify the most meaningful themes from participants’ reports. The analysis of the transcripts yielded three main themes. The first theme refers to participants’ awareness of the emotional component in managing the child’s fever and the challenges this component presents. The second theme refers to the risk of overtreating when the child’s right to be sick is not recognized and respected. The third theme refers to the importance of the relational component, showing how a solid therapeutic alliance with the healthcare team helps caregivers develop self-confidence in managing the child’s fever. This study contributes to advance our understanding of fever phobia and to a better conceptualization and operationalization of this phenomenon.

*Conclusion*: Our results point out to the importance of going beyond a knowledge gap paradigm and recognizing both the emotional and the relational component of fever phobia, the former being entrenched in latter, that is, the unique relationship caregivers establish with their child’s paediatrician and the medical assistant.

**Table:** 

**What is Known:** *• Research on fever phobia has predominantly focused on the role of fever misconceptions in triggering anxiety and impeding a proper fever management, in terms of both concept and operationalization, with little attention to the relational component of this phenomenon.*	
**What is New:** *• Our results point out to the importance of recognizing the emotional component of fever phobia, beyond its declarative and procedural knowledge dimensions. They also suggest that overtreating is not necessarily and not only the result of a phobia but also of a particular conception of health and the relational component of this phenomenon, which is entrenched in the unique relationship caregivers establish with their child’s paediatrician and the medical assistant.*

**Supplementary Information:**

The online version contains supplementary material available at 10.1007/s00431-022-04704-4.

## Introduction

In childhood, fever is a common sign, which is often associated with pain and discomfort [1]. Although it may occasionally be caused by a potentially life-threating infection, most cases are caused by benign, self-limiting infections [2, 3]. Despite a plethora of clinical guidelines [4, 5] on the correct symptomatic management of fever [6, 7], several studies have reported inaccurate knowledge about this symptom and inappropriate management behaviours among caregivers [8]. There is evidence that caregivers’ management of fever is largely influenced by unrealistic and unwarranted concerns about the potential harm that elevated body temperature can cause, a phenomenon commonly referred to as fever phobia [9]. Fever phobia appears to be associated with caregivers’ low educational or socioeconomic backgrounds, low income, lack of health insurance, history of febrile seizures, young maternal age and belonging to certain ethnic or cultural groups [10–16]. Finally, studies showed that healthcare professionals such as paediatricians, nurses and pharmacists may hold different views and misconceptions about fever and its management, with many believing that fever in itself can lead to serious complications [17–19]. Thus, they may be contributing to fever phobia by presenting mixed and inaccurate messages about fever and its treatment [19, 20]. Switzerland is not fever phobia free. A survey conducted in the three main Swiss language regions showed that the majority of caregivers hold fever phobic beliefs, resulting in widespread improper fever management [15].

Research on fever phobia has predominantly focused on the role of fever misconceptions in triggering anxiety and impeding a proper fever management, in terms of both concept and operationalization, with little attention to the relational component of this phenomenon (i.e., the quality of the relationship between parents and providers and the role that an “abnormal” interaction could have in shaping fever management practices). However, research has highlighted the importance of patient–provider relationships in influencing parents’ adherence to paediatric health and safety guidelines [21]. For this reason, we conducted a pilot study to explore and describe fever-related knowledge (including beliefs about fever, its cause and its consequences), experience and behaviour among a sample of caregivers, paediatricians and their medical assistants.

## Methods

### Study design

In order to explore and describe fever-related knowledge, experience and behaviour among caregivers and healthcare professionals, we employed a qualitative study design with semi-structured, one-to-one interviews. Qualitative methods are considered valuable in providing rich descriptions of complex phenomena and illuminating the experience and interpretation of events by actors with widely differing stakes and roles [22]. We conducted the study in the Canton of Ticino in Southern Switzerland, where Italian is spoken, between April 1, 2020, and June 30, 2021. The method and reporting follow the Consolidate Criteria for Reporting Qualitative Research (COREQ) [23].

### Recruitment and sample

We employed a purposive sampling strategy to identify potential paediatricians employed in private healthcare facilities in the Canton of Ticino. Through contact with the paediatricians, we aimed at reaching a sample of 20 participants: 5 paediatricians, 5 medical assistants and 10 caregivers. All participants had to be resident in the Canton of Ticino and be fluent in Italian. Parents had to have at least one child between the ages of 0 and 3 years. The recruitment process is explained in Appendix 1. Due to initial difficulties in reaching the target sample of caregivers, these latter were recruited by direct contact with paediatricians or using flyers and posters in paediatric offices.

### Data collection

We conducted individual interviews either in person or by phone between October 2020 and February 2021. In-person interviews were conducted at mothers’ home or the University of Lugano, while it was the paediatrician’s office for paediatricians or medical assistants. More information on data collection can be found in Appendix 1.

Each interview was audio-recorded after informed consent and transcribed verbatim. During the interviews, we collected data on mothers’ socio-demographic characteristics such as gender, year of birth, occupation, nationality, district of residence and number of children, as well as data on paediatricians’ and their medical assistants’ gender, age and years of work experience.

We developed a semi-structured interview grid in Italian, accounting for the specificities of caregivers, on one side, and paediatricians/medical assistants, on the other. The grid was based on existing literature describing previous qualitative investigations of fever phobia [24–26]. Insights from participants progressively informed the addition of new questions to the interview grid, see Appendix 2 for the exhaustive list of questions used during the interviews.

### Data analysis

We employed an inductive–deductive analysis of the transcripts to identify the most meaningful themes from participants’ reports. Three coders (FM, MF and IF) independently conducted an analysis of the transcripts in the original language (Italian) following the six-stage comprehensive thematic analysis approach developed by Braun and Clarke [27]. The analysis is detailed in Appendix 1. Thematic analysis was considered a valid approach to our data, because we adopted an in-depth interviewing style and reports—despite limited in the number—were rich enough to allow the extraction of a variety of themes. We presented categorical variables as absolute frequency and percentage, whereas continuous data as median and range.

## Results

### Participants’ characteristics

The final sample included 19 participants, namely, 10 mothers, 5 paediatricians and 4 paediatricians’ medical assistants. Most mothers were Swiss (*n* = 6), were a resident in the district of Lugano (*n* = 9), had an academic degree (*n* = 6) and were employed (*n* = 9). Their median age was 37.5 years (range = 30–39). They had an average number of 2.4 children (compared to the average of 1.36 and 1.45 children among Swiss and foreign national mothers born in Switzerland, respectively) [28]. Most paediatricians were men (*n* = 4), Swiss nationals (*n* = 4) and a resident in the Lugano district (*n* = 3). Their years of work experience was median = 23, and they had 2.2 children each on average. Their median age was 58 years (range = 41–65). All medical assistants were women and Swiss nationals (*n* = 4). They had an average of 1.75 children each and 25.5 median years of work experience. Their median age was 48.5 years (range = 37–52), see Table 1 for an overview of participants’ characteristics and Table 2 for exemplary quotes from participants’ interviews.

**Table 1:** Characteristics of study participants (*N* = 19)

	Parents (*N* = 10)	Pediatricians (*N* = 5)	Medical assistants (*N* = 4)
Female gender	*N* = 10 (100%)	*N* = 1 (20%)	*N* = 4 (100%)
Age (years)	Median = 37 (range = 30–39)	Median = 58 (range = 41–65)	Median = 48 (range = 37–52)
Number of children	*M* = 2.4	*M* = 2.2	*M* = 1.75
District of residence			
Lugano	*N = 9 (90%)*	*N = 3 (60%)*	*N = 2 (50%)*
Mendrisio	*-*	*-*	*N = 1 (25%)*
Locarno	*N = 1 (10%)*	*N = 1 (20%)*	*N = 1 (25%)*
Chiasso	*-*	*N = 1 (20%)*	*-*
Swiss nationality	*N* = 6 (60%)	*N* = 4 (80%)	*N* = 4 (100%)
Years of experience*	-	Median = 23 (range = 9–33)	Median = 25 (range = 21–31)
Education			
Secondary school	*N = 4 (40%)*	-	*N = 4 (100%)*
University	*N = 6 (60%)*	*N* = 5 (100%)	*-*
Currently employed	*N = 9 (90%)*	-	*-*

^*^Years of experience are counted since obtaining medical degree and end of nursing apprenticeship, respectively

**Table 2:** Exemplary quotes from participants’ interviews

Theme	Quote	#
The emotional component of fever phobia	I have to say that up to 39 I don’t get worried. The paediatrician has always told me that 38° is not fever when it comes to young children, so I don't panic. On the other hand, my husband has a hard time dealing with the hot condition of fever. […] When my older daughter has fever, she can't sleep, so I keep reassuring her, and when she falls asleep once the medicine kicks in, I feel calmer. Her dad, on the other hand, has a big crisis every time the girls are sick, and then I have to reassure him too (Mother, 39 years old, two children)	1
	I think it is very difficult for someone to help parents to be truly informed. We can inform, but what I see in daily practice is that I repeat exactly the same things over the years, for the same people. So it is very difficult to remove that fatalism, those fears that parents may have with respect to that number written on the thermometer. It’s hard to help them to develop a certain capacity to reason about what the child is like. Parents lost sight of their children’s real status. Close your eyes: what do you feel? Do that, rather than looking at the thermometer and thinking it’s bad because the temperature is high! (Male paediatrician, 64 years old, 30 years of experience)	2
	It is very different when you are in the medical practice. You experience emotions but you are more cold-blooded than with your son, because other emotions emerge… When I feel less rational I tell myself “Stop it, don't panic with your daughter”! Or I try to remind myself that I know these things (Female medical assistant, 46 years old, 21 years of experience)	3
	Experience teaches you. When you experience the first fevers of your children, you immediately call the paediatrician and ask what to do. You ask him/her if you have to bring them for a visit. Now I tend to observe how things go. I call the paediatrician and I evaluate the situation only after two days of fever (Mother, 39 years old, three children)	4
	Answers are not always the same. Recently, I met a mother of several children who had her 10-day-old baby with fever, and said “I'll wait for the next day”. But with a baby of ten days it’s unthinkable to wait for the next day. So, those automatisms that she used for the older ones did not work with this one (Female paediatrician, 47 years old, 15 years of experience)	5
The child’s unspoken right to be sick is at risk	The child’s feverish state must be treated with respect for the child. Saying that fever starts at 38° automatically allows the adult to dispose of the child as he or she wishes. Sometimes parents react to fever by giving the child something, so that at least she does not feel sick. No, you can’t do it for your convenience. Parents need to be educated to manage this situation in a clever, not an opportunistic way (Mother, 37 years old, five children)	6
	[Parents] want to lower the child’s fever at all costs. There is a discrepancy between private practices and the hospital. In the hospital, I always have the impression that drugs are given at maximum dosages because the child is very sick. But the message that is passed on to the mothers is that they should go on giving these drugs to their child for several days, even when the child feels better (Female paediatrician, 47 years old, 15 years of experience)	7
	We have very little patience today: if a child coughs, we want to give her a medicine straight away to stop the cough. A child no longer has the right to be sick and has to always be healthy (Female medical assistant, 51 years old, 20 years of experience)	8
	You know, nobody talks about the sick child and there are so many obscure points regarding being a parent. Nobody talks to you about the difficulties that can exist with breastfeeding, for example, which I experienced only after having five children. If you don't have the right people by your side, you can have so many negative experiences (Mother, 39 years old, five children)	9
	Every now and then the management would call you and ask you “why did you talk about these things”? Cause maybe a dad had complained because his wife was frightened by this topic… Every mom and every dad gets it in a way different. Okay, the topic of fever [seems straightforward]. But you have to be careful because if the child has meningitis, he may only have 38.5° of fever, but the general condition may not be that good. You then inform the parent, and there are those who take it as good information, and those who take it in another way, like “this guy is crazy”. Here, in my opinion, here is where we must be careful (Male paediatrician, 58 years old, 23 years of experience)	10
The relational component of fever phobia	You basically call him [the paediatrician] to hear a friendly voice, to hear that everything is fine, even if deep down you know it is not that serious (Mother, 35 years old, two children)	11
	My role is precisely that of trying to calm the mother about the benignity of the symptoms and that the smaller the child, the more I have to be careful because the child's cooperation is a little less good. So the first thing is to reassure the mother on the benignity of the fever, because sometimes mothers are alarmed. So this is my first role, a sort of a firefighter (Male paediatrician, 65 years old, 33 years of experience)	12
	It is so important to have someone like me or my colleague, someone who they trust more, a closer relationship, someone who they feel more comfortable with. It’s always the same, it’s hard to explain. It comes natural to me because parents feel reassured (Female medical assistant, 51 years old, 20 years of experience)	13

### Extracted themes

The analysis of the transcripts yielded three main themes. The first theme refers to participants’ awareness of the emotional component in managing the child’s fever and the challenges this component presents. The second theme refers to the risk of overtreating when the child’s right to be sick is not recognized and respected. The third theme refers to the importance of the relational component, showing how a solid therapeutic alliance with the healthcare team helps caregivers develop self-confidence in managing the child’s fever.

### The emotional component of fever phobia

Almost all participants recognized the importance of emotions in the management of children’s fever. Mothers explained that having appropriate and accurate information on fever and its management is not enough to remove the anxiety associated with managing a feverish child. Several mothers reported that you may know exactly what to do, but still manage the child’s fever with a high degree of stress and anxiety (#1). All paediatricians agreed that the emotional component is the most important component in the management of children’s fever. One paediatrician added that this constitutes a major obstacle when it comes to educate caregivers about the management of their children’s fever and the importance of observing the child’s status beyond the body temperature (#2). This view was shared by medical assistants too, who recognized the difficulty to control one’s emotions, even for someone like them who has all the appropriate information and skills to manage the child’s fever (#3). According to some mothers, experience is key in decreasing the anxiety associated with the child’s fever (#4). However, one paediatrician reported that experience can also guide caregivers towards dangerous decisions (#5).

### The child’s unspoken right to be sick is at risk

Some mothers recognized that the appropriate approach to fever management requires “respect” for the child (#6). This view was shared by paediatricians, who unanimously reported that children have a “right to be sick”. One paediatrician added that hospitals have been responsible for leading caregivers and paediatricians towards an attitude that ignores this right (#7). The view that fever is treated with impatience and that the child’s right to be sick is often disregarded is shared by medical assistants as well (#8). According to some mothers, the reason why fever is managed with anxiety is that no one talks about the sick child during birth preparation courses and caregivers find themselves unprepared and without the appropriate support (#9). However, a paediatrician explained that even when paediatrician talk to caregivers about the possibility of the child experiencing fever or illness, they must be careful as the topic is often received negatively by caregivers (#10).

### The relational component of fever phobia

Mothers reported that a major role in managing their children’s fever is played by the relationship they established with the child’s paediatrician and the paediatrician’s assistant. Mothers who reported a positive experience and a high degree of trust referred to an alliance with the healthcare team based on an emotional approach (#11). Paediatricians confirmed that they saw their role as that of emotional companions, who have to first of all reassure and calm caregivers down (#12). Medical assistants shared this view and added that such an emotional approach is key in establishing trust, which in turn helps caregivers become more confident in managing their children’s fever. One assistant reported that it is a natural attitude, she is more open because she wants mother to feel reassured (#13).

## Discussion

In this pilot study, we qualitatively explored fever-related knowledge, experiences and behaviour among a small sample of caregivers (mothers), paediatricians and their medical assistants in the Canton of Ticino, Southern Switzerland. The study represented a small-scale test of the interview grid that we plan to use on a larger scale. A first finding is that participants unanimously reported that emotions played a central role in caregivers’ management of their children’s fever. Other studies highlighted the “phobic” component in the management of children’s fever. In qualitative studies conducted on this topic, caregivers acknowledged feelings of concern, fear, feelings of being overwhelmed, freezing up and a sense of relief once the fever was controlled [25]. However, most quantitative studies operationalized fever phobia in terms of three main dimensions: knowledge about fever definition (and possible consequences), its measurement and management. Fear is an emotion of anticipation that is triggered when we perceive a situation that is at risk for our safety and/or the safety of others [29]. To prepare the body to face this danger, stimuli can evoke “freeze, flight, fight, fright” reactions [30] or “tend-and-befriend” responses (such as turning to others for help or social support, or making a situation less tense, dangerous, or uncomfortable in some way) [31]. Phobia in this sense has been neglected by previous studies and simply assumed as a consequence of not having adequate fever literacy. Our findings that emotions and knowledge are not necessarily associated are aligned with research suggesting that educating caregivers may help reduce their uncertainty and anxiety, improve coping and fever management and lead to better medication management, less antibiotic prescription and fewer visits to providers [19, 32–35]. Educational efforts have targeted not only caregivers but also clinicians, nurses, pharmacists, shopkeepers and medical students [36]. However, a recent meta-analysis showed that, despite efforts to improve caregivers’ fever literacy and management, anxiety persists [37]. Our participants highlighted the importance of an emotion-based approach at the core of their relationship with the paediatrician and the medical assistant. Qualitative evidence highlights caregivers’ need for professional, consistent information and reassurance from an expert, their desire for feeling listened to and in control, and the role of their experience, of the internet and of one’s social network as information sources [24, 26, 38–42]. Agency (maintaining a sense of control over future events) allows caregivers to endure hardship, deal with their child’s illness and remain actively engaged with the child’s future wellbeing and the desired outcome. Paediatricians and medical assistants seem to be in the best position to preserve and sustain this agency, due to the long-term, intense, regular and complicity-demanding relationship they establish with caregivers.

A second, related finding is that a proper management of fever should be grounded in the idea that the child has a right to be sick, that this right should be honoured, and the child be treated with respect. Rather than in opposition to a right to be healthy, the right to be ill has been defined as both the “freedom to be sick” (e.g., the right to refuse to start or continue a therapy) and as the right to enjoy the same quality of life as anyone else [43]. Our participants, however, described the child’s right to illness as her right to not be overtreated, to experience fever as the natural expression of the underlying disease, and for her body to fight the disease by itself as much as possible. These findings could be seen as an expression of recent movements that have been particularly popular in Switzerland that pushed towards healthism and a less medicalized approach to illness [44].

Our results have a number of implications for the conceptualization of fever phobia and for clinical practice and training. Traditionally, this phenomenon has been conceptualized as an individual construct (with focus on caregivers) and measured by assessing caregivers’ misconceptions in terms of knowledge about fever definition, measurement and management [9]. Our results highlight the importance of the emotional and relational component in managing children’s fever, which has so far been operationally neglected by empirical investigations of this phenomenon. Healthcare professionals should be made aware of the importance of caregivers’ emotions during fever management and be trained on how to recognize and address them and constantly focus on developing and maintaining a trustworthy relationship with caregivers. Our results suggest that fever phobia may be only one of the dimensions of fever overtreatment. Therefore, instead of referring to fever phobia, we should rather refer to fever overtreatment, a neutral term which can include multiple motivations.

Interestingly, our participants referred to caregivers who tend to overtreat their children’s fever in peculiar ways, framing this approach in a negative way as a form of exaggeration and impatience. This result raises the question whether fever overtreatment is connected to caregivers’ working status. Are working caregivers who cannot “afford” to let the fever run its course and monitor the child at the same time more likely to engage in unorthodox fever management practices? If this association was found to be true, it would be a mistake to address this phenomenon as fever phobia, as it would represent a strategy to manage the child’s illness while at the same time honouring one’s work commitments despite knowing what the right thing to do is.

### Limitations

Several limitations to this study are worth being mentioned. First, we cannot exclude that some participants reported their views in a way that would be considered socially desirable. However, we adopted a non-judgmental interviewing style, reassured participants about our commitment to confidentiality and encouraged them to share their views, experiences, and concerns openly. Second, individuals that were invited but who chose not to participate in the study and those that took part to it may not be equivalent and have very different views. Third, we do not know whether the lack of male participants is because they rarely presented at the paediatricians’ offices and could not be invited or they were invited and refused. In both cases, their absence suggests that mothers represent the health gatekeepers of the family (even when it comes to participate to research). Fourth, despite our efforts in the recruitment, we managed to reach a limited sample that is not diverse in terms of education and type of settlement (most mothers had an academic degree and were resident in a urban area). Finally, being this a qualitative study with a small sample, our goal was not to generate results that could be generalized to other geographic contexts, particularly countries with a different healthcare system than Switzerland (for which intent a quantitative design is needed). The large majority of Swiss-based paediatricians who are not employed in a hospital work alongside one or more medical assistants and directly bill the child’s caregivers for each consultation according to its nature and length. In other healthcare systems (e.g., Italy), providers’ income depends on the number of patients and not on how many consultations they offer. This has implications for both the length of the encounter and for hiring an assistant. Attention must be paid to generalize the results within Switzerland, too. The average number of children among the mothers who participated in this study was higher than the total fertility rate of Swiss and foreign national mothers born in Switzerland in 2020. These limitations need to be interpreted in light of the significant resources we invested in the recruitment phase. Indeed, the small and the hardly diverse sample we managed to interview suggests that this is a hard-to-reach population. However, the qualitative nature of this investigation was key to generate meaningful insights into the phenomenon of fever phobia that would otherwise be hard to capture using a different methodology.

## Conclusions

Our results point out to the importance of recognizing the emotional component of fever phobia—beyond its declarative and procedural knowledge dimensions—that overtreating is not necessarily and not only the result of a phobia but also of a particular conception of health and the relational component of this phenomenon, which is entrenched in the unique relationship caregivers establish with their child’s paediatrician and the medical assistant. Data on caregivers’ and healthcare professionals’ beliefs, experiences and needs regarding fever and fever management could inform the development of complex, multicomponent interventions to reduce caregivers’ fever phobia and improve fever management.

## Supplementary Information

Below is the link to the electronic supplementary material.

Supplementary file1 (DOCX 14 KB)

Supplementary file2 (DOCX 20 KB)
