# Supplementary material for: New insights into fever phobia: a pilot qualitative study with caregivers and their healthcare providers
Source: Eur J Pediatr. 2022 Nov 29;182(2):651–9. doi: 10.1007/s00431-022-04704-4 (PMC9899170; doi:10.1007/s00431-022-04704-4)
Supplement: Supplementary file 1 — Supplementary file1 (DOCX 14 KB) [file 431_2022_4704_MOESM1_ESM.docx]

Appendix 1.

Recruitment process

We sent a letter to ten paediatricians with diverse clinical experience identified by GDS and MGB, and invited them to participate in the study. The letter invited them to participate in an in-person or telephone interview on the topic of fever, and asked them to identify 3-5 caregivers (e.g., mothers, fathers, or both) of children younger than three to be included in the study. Together with the letter, we provided a detailed explanation of the study aim and procedures, and a printed sheet where paediatricians or their medical assistants could record the phone number of those caregivers accepting to be contacted by our team for an in-person or telephone interview. A month after the invitation letter, we contacted the paediatricians to ask them whether they would like to participate in the study andif they had identified potential participants among their patients’ caregivers. Among the ten paediatricians contacted, 5 decided to participate in the study and provided a list of 10 mothers. Following the first five interview, we extended the study to medical assistants. In Switzerland, medical assistants complete administrative and clinical tasks in offices of paediatricians (e.g., they record patient history and personal information, measure vital signs, help physicians with patient examinations, provide information and advice). Four medical assistants (working with four of the paediatricians who adhered to the study) accepted to be interviewed. To improve recruitment of caregivers, flyers and posters were distributed in the offices of the paediatricians who accepted to be part of the study. The flyers and posters invited potential participants to make direct contact with the research team. The final sample included a total of 19 participants: 5 paediatricians, 4 medical assistants, and 10 mothers of at least one child aged ≤3 years.

Data collection

The interviews lasted approximately one hour and were conducted by three members of the research team (FM, IF, and MF), either individually or in pair (e.g., IF and FM, together). During the interview, participants were not aware of other participants’ answers. MF (she/her) is a researcher with extensive experience in qualitative health research and with a record of academic appointments in the teaching of qualitative research methods; FM (she/her) is a social worker with a 3-year experience in qualitative health research; IF (she/her) is a trained psychologist with a 3-year experience in qualitative health research.

Data analysis

The coders read the transcripts several times to familiarize themselves with the content, highlighting important quotes, and identifying different themes. Subsequently, they identified relationships between different themes, and discussed convergences and divergences among them. Disagreement between the coders was resolved through discussion and by constantly referring to the corpus of data. A group meeting among all the authors took place after this initial analysis to present the preliminary study results. After the themes were discussed and divergences among team members were resolved to reach consensus on the final themes and sub-themes, the three coders (FM, MF and IF) employed a deductive analysis of the data, searching quotes that were representative of the agreed-upon themes.
