# Supplementary material for: New insights into fever phobia: a pilot qualitative study with caregivers and their healthcare providers
Source: Eur J Pediatr. 2022 Nov 29;182(2):651–9. doi: 10.1007/s00431-022-04704-4 (PMC9899170; doi:10.1007/s00431-022-04704-4)
Supplement: Supplementary file 2 — Supplementary file2 (DOCX 20 KB) [file 431_2022_4704_MOESM2_ESM.docx]

Appendix 2. Interview questions

Interview questions targeting caregivers:

| **Topic(s)** | **Question(s)** |
| --- | --- |
| Definition of fever and of high fever, its causes and role during the child’s illness | How would you define fever (e.g. temperature)? What is its role in the child’s illness? What causes fever? |
| Definition of child’s discomfort | From what can you tell that your child does not feel comfortable? |
| Experience in measuring and managing the child’s illness in general and fever in particular, including both evidence-based and non-evidence-based physical and drug treatments | How do you measure your child’s temperature? What do you do, in general, when your child has fever? |
| Perceived barriers to and enablers of a correct measurement and management of fever | What prevents you from adhering to the recommended medication (if applicable)? What helps you stick to what the pediatrician recommends? |
| Positive and negative feelings associated with the measurement and management of the child’s disease in general and fever in particular | How do you feel when your child is not well? How do you feel when your child has fever? |
| Expectations from the pediatrician | Ideally, how would you like your child’s pediatrician to manage your child’s fever? |
| The process by which information on fever measurement and management is acquired, developed and enhanced (including past and current information sources, and the social context caregivers have been exposed to through their life and, particularly, during pregnancy (if applicable) and child’s early life (first three months) | Think about the information you have on fever management. How did you obtain this information? When? From whom? |
| Preferences and expectations regarding the communication of information about fever measurement and management (including preferences on the content, the format, the sender, the channel, the timing, etc.) | Imagine you could design a way to send information on fever management to soon-to-be parents: what would you tell them? How? Through which channels? At what point in terms of their child’s age? |
| Preferences regarding engagement in the design and implementation of a future intervention to help caregivers measure and manage their child’s fever in a safe, guidelines-compliant and anxiety-free manner | If you participated in the design of an intervention to help caregivers measure and manage their child’s fever in a safe, guidelines-compliant and anxiety-free manner, how would you like to be involved? |

Interview questions targeting pediatricians and medical assistants

| **Topic(s)** | **Question(s)** |
| --- | --- |
| Definition of fever and of high fever, its causes and role during the child’s illness | How would you define fever (e.g. temperature)? What is its role in the child’s illness? What causes fever? |
| Definition of child’s discomfort | From what can you tell that a child does not feel comfortable? |
| What should count as fever literacy | What information do you think caregivers and healthcare professionals should necessarily have to be considered fever literate? Which (e.g. numeracy) skills count as fever literacy? |
| Strategies to manage caregivers’ expectations | How do you manage caregivers’ expectations in relation to their child’s fever, when these are not in line with evidence-based practice?  What is your experience with caregivers of different cultural backgrounds?  Have you noticed any differences? If so, of what kind? |
| Personal experience with measuring and managing own child’s/children’s fever (if applicable), including practices, feelings, information sources, and barriers to/enablers of appropriate practices | How do you measure your child’s temperature? What do you do, in general, when your child has fever? Think about the information you have on fever management. How did you obtain this information? When? From whom? How do you feel when your child is not well? How do you feel when your child has fever? |
| Past and current education and counselling practices targeting caregivers and other healthcare professionals concerning fever measurement and management strategies (including physical and drug treatment) | What do you recommend caregivers and other healthcare professionals about fever measurement and management? How do you recommend it? |
| Perceived barriers to and enablers of a correct management of fever among caregivers | What prevents caregivers from adhering to the recommended medication? What helps them stick to what you or other healthcare professionals recommend? What kind of differences have you noticed (if any) in terms of caregivers’ adherence to your recommendations? |
| Desirable features of a possible, future intervention aimed at reducing fever-phobia and improve fever management behaviors among caregivers | Imagine you could design a way to send information on fever management to soon-to-be parents: what would you tell them? How? Through which channels? At what point in terms of their child’s age? |
| Efficient and effective ways in which to engage caregivers in the design and implementation of the future intervention | If you were to design an intervention to help caregivers measure and manage their child’s fever in a safe, guidelines-compliant and anxiety-free manner, how would you involve them in the development process? |
